# ODDM: Integration of SMOTE Tomek with Deep Learning on Imbalanced Color Fundus Images for Classification of Several Ocular Diseases

**DOI:** 10.3390/jimaging11080278

**Published:** 2025-08-18

**Authors:** Afraz Danish Ali Qureshi, Hassaan Malik, Ahmad Naeem, Syeda Nida Hassan, Daesik Jeong, Rizwan Ali Naqvi

**Affiliations:** 1 Department of Computer Science, National College of Business Administration & Economics Lahore, Multan Sub Campus, Multan 60000, Pakistan; afraz122310290@ncbaemultan.edu.pk (A.D.A.Q.); campusmanagerwtc@ncbae.edu.pk (H.M.); 2 Department of Computer Science, NFC Institute of Engineering and Technology, Multan 60000, Pakistan; ahmad.naeem@nfciet.edu.pk; 3 Department of Business and Computing, Ravensbourne University, London SE10 0EW, UK; syedanida.hassan@students.rave.ac.uk; 4 Division of Software Convergence, Sangmyung University, Seoul 03016, Republic of Korea; 5 Department of AI and Robotics, Sejong University, Seoul 05006, Republic of Korea

**Keywords:** ocular disease, eye disease, AMD, diabetic retinopathy, deep learning, CFI

## Abstract

Ocular disease (OD) represents a complex medical condition affecting humans. OD diagnosis is a challenging process in the current medical system, and blindness may occur if the disease is not detected at its initial phase. Recent studies showed significant outcomes in the identification of OD using deep learning (DL) models. Thus, this work aims to develop a multi-classification DL-based model for the classification of seven ODs, including normal (NOR), age-related macular degeneration (AMD), diabetic retinopathy (DR), glaucoma (GLU), maculopathy (MAC), non-proliferative diabetic retinopathy (NPDR), and proliferative diabetic retinopathy (PDR), using color fundus images (CFIs). This work proposes a custom model named the ocular disease detection model (ODDM) based on a CNN. The proposed ODDM is trained and tested on a publicly available ocular disease dataset (ODD). Additionally, the SMOTE Tomek (SM-TOM) approach is also used to handle the imbalanced distribution of the OD images in the ODD. The performance of the ODDM is compared with seven baseline models, including DenseNet-201 (R_1_), EfficientNet-B0 (R_2_), Inception-V3 (R_3_), MobileNet (R_4_), Vgg-16 (R_5_), Vgg-19 (R_6_), and ResNet-50 (R_7_). The proposed ODDM obtained a 98.94% AUC, along with 97.19% accuracy, a recall of 88.74%, a precision of 95.23%, and an F1-score of 88.31% in classifying the seven different types of OD. Furthermore, ANOVA and Tukey HSD (Honestly Significant Difference) post hoc tests are also applied to represent the statistical significance of the proposed ODDM. Thus, this study concludes that the results of the proposed ODDM are superior to those of baseline models and state-of-the-art models.

## 1. Introduction

The number of cases of ocular diseases (ODs) that can impair vision, such as trachoma, diabetic retinopathy (DR), cataracts, and age-related macular degeneration (AMD), has significantly increased in the past 20 years. According to a report by the World Health Organization (WHO), there are more than 2.2 billion individuals with vision impairments in the world. At least 45 percent of these incidents are unresolved or could have been avoided [1]. The primary causes of vision loss and blindness are cataracts, trachoma, and refractive problems left untreated (such as myopia, astigmatism, hypermetropia, and presbyopia). The WHO estimates that 10.6 million individuals have been diagnosed with trachoma, approximately 18 million people are bilaterally blind from cataracts, and more than 153 million are affected by chronic refractive problems that affect vision [2]. Furthermore, studies [1,2,3] revealed that AMD is the leading cause of blindness globally, accounting for 8.7% of cases (or 3 million individuals) of blindness, especially in developed nations. By 2040, it is anticipated that there will be 10 million instances [1,2,3,4,5,6]. Recent studies [4,5] also showed that 4.8% of the 37 million cases of blindness reported globally (e.g., 1.8 million people) are caused by DR.

In ophthalmology, ocular fundus imaging [7] is frequently used as a practical and affordable method of screening for retinal abnormalities and tracking the advancement of disease. Retinal images have excellent inter- and intra-examination agreement, sensitivity, and specificity when compared to in-person ophthalmologist examinations. Thus, in many clinical scenarios, retinal images can be used instead of ophthalmoscopy. Even in the absence of pupillary dilatation, high-quality retinal images are now more easily obtained because of developments in optical fundus imaging. Fundus cameras have several benefits, and because of the floodlight’s single-flash exposure, they are practical for patients. Furthermore, they do not affect the quality of the image in certain scenarios, such as a decrease in degeneration in cases of cataracts.

**Figure 1:**
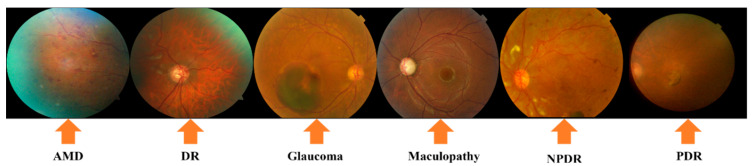
A few samples of CFIs of ODs.

However, detecting eye disorders comes with several difficulties. First off, many common ODs like DR, AMD, and cataracts proceed without any obvious symptoms at first, making accurate early diagnosis challenging [8]. Secondly, it could take a while for doctors to determine the patient’s ailment. Third, the diagnosis requires specialists who are not always available. Fourth, even with the benefits that ocular fundus imaging offers, obtaining enough precise fundus images can occasionally be challenging, particularly for certain uncommon fundus illnesses [9]. The main reason for this is that it can be difficult to distinguish between the generated fundus pictures and eye anatomies due to their low contrast [10]. As a result, ophthalmologists may not accurately detect every indication of an eye condition. Sample images of ODs are presented in Figure 1.

OD rates are increasing at an alarming rate, although if patients are found and treated early, their odds of losing sight are better than 95% [8,9,10,11]. This motivates us to develop a model for the early detection of ODs to cure humans of blindness. Thus, this study introduces a custom multi-classification model, named the deep learning-based OD detection model (ODDM), based on a CNN, that classifies the normal (NOR) case and six types of ocular disorders, including AMD, DR, maculopathy (MAC), PDR, NPDR, and glaucoma (GLU), using CFIs.

Artificial intelligence (AI) approaches have been proposed to automate the process of OD detection to solve the aforementioned issues [11]. Machine learning (ML) techniques have been frequently used for the diagnosis of eye diseases [11,12]. Ocular diagnostic systems built with SVM and other traditional classifiers [13], in addition to K-nearest neighbors (KNNs) [14], showed strong performance on small datasets but poor performance on large datasets. Because OD detection is more difficult and specialized, these approaches might not be appropriate. Moreover, feature extraction has been performed manually in traditional ML methods. Recently, deep learning (DL) has emerged as the industry standard for computer vision technologies. Creating novel medical image processing algorithms to aid in health identification and diagnosis has drawn a lot of research interest [15,16,17,18,19,20,21]. DL approaches do not require lesion segmentation or labor-intensive feature identification and processing, in contrast to traditional ML algorithms [9,11]. CNNs have revolutionized the way basic computer vision and image processing problems like segmentation and classification are solved [22,23,24]. To evaluate the proposed ODDM, a publicly available benchmark OD [25] dataset is used in this study. An OD dataset has the issue of class imbalance; therefore, SMOTE Tomek (SM-TOM) is used to overcome this issue. The reason for choosing SM-TOM over other resampling methods is that it not only balances the OD dataset but also improves model performance [13] by eliminating noise [16] and refining decision boundaries [8]. Additionally, it also creates synthetic examples that interpolate between the various instances of minority features, thus minimizing the problem of overfitting due to random duplication. The capacity of generating more diverse and general samples makes it perform better on imbalanced data. Research studies [26,27,28,29] show significant performance in the binary classification of ODs; however, no evidence has been found that DL models classify several types of ODs such as AMD, DR, MAC, PDR, NPDR, and GLU. Additionally, the ODDM was compared to 06 baseline classifiers including DenseNet-201 (R_1_) [30], EfficientNet-B0 (R_2_) [31], Inception-V3 (R_3_) [32], MobileNet (R_4_) [33], Vgg-16 (R_5_) [34], Vgg-19 (R_6_) [35], and ResNet-50 (R_7_). The major contributions of this study are stated below:

1. Seven different types of ODs, including NOR, AMD, DR, MAC, PDR, NPDR, and GLU, are classified using the proposed ODDM. The proposed ODDM has the ability to extract the dominant features from CFIs that can be helpful in the accurate classification of ODs. Furthermore, this study also simplifies the proposed ODDM by reducing the number of trainable parameters to obtain a significant classifier.
2. SM-TOM is used to handle the imbalance class issue of the OD dataset, and the Grad-CAM heatmap technique is employed to highlight the infected region that occurred in the eye due to ODs.
3. Ablation experiments are performed to evaluate the effectiveness of the proposed ODDM, and the ANOVA and Tukey HSD (Honestly Significant Difference) post hoc tests are used to show the statistical significance of the proposed ODDM. Also, the proposed ODDM obtained 97.19% accuracy, which is superior to that of modern state-of-the-art (SOTA) approaches.

This study contains the following sections: Section 2 presents the modern literature that uses AI methods for the classification of ODs using different medical imaging modalities. Section 3 presents the comprehensive details of the dataset description, SM-TOM, the proposed ODDM, and performance evaluation matrices. In Section 4, the experimental results are discussed. Lastly, this study is concluded in Section 5.

## 2. Literature Review

Modern ML and image processing methods dominate the DR detection literature. Previous research studies used image processing techniques to pre-process fundus images and extracting features. These resulting features of CFIs were then classified into the respective eye disease classes using an AI approach. Table 1 presents the modern literature that used AI methods for the classification of ODs. One study [36] proposed a pre-trained multi-class classification model for several ocular disorders. This model classifies fundus images into several OD categories using a CNN and pre-trained classifiers. Peking University’s Ocular Disease Intelligent Recognition collection includes annotated images labeled as NOR, DR, GLU, myopia, AMD, and other disorders. Two pre-trained models, ResNet-50 and Vgg-16, were proposed for the identification of ODs. The ResNet-50 and Vgg-16 models were used to combine feature vectors from the right and left fundus images of the eye, and they achieved an accuracy of 92.35%.

Vidivelli et al. [37] proposed a multi-label DL model named CataractNetDetect for the classification of cataracts from pairs of CFIs. The proposed CataractNetDetect model demonstrates significant outcomes and achieves a 97.90% AUC. Li et al. [38] used two different CNN-based pre-trained models for the classification of ophthalmological disorders. They trained and tested these models on a publicly available benchmark ODIR dataset. Additionally, the performance of the two optimizers, Stochastic Gradient Descent (SGD) and Adam, was also observed. The highest testing accuracy of 89.64% was achieved by the MobileNet model with the Adam optimizer.

Rubina et al. [39] used a Vgg-16 model for the classification of DR. They divided DR into two classes including mild multi-class diabetic eye diseases (DEDs) and multi-class DED. Vgg-16 achieved a classification accuracy of 88.30% and 85.95% on multi-class DED and mild multi-class DED, respectively.

One study [40] designed a deep convolutional neural network (DCNN) model to classify retinal fundus images into binary categories. The authors used the APTOS 2019 dataset to train and test their proposed model and achieved a significant classification accuracy of 90.35%. Pawar et al. [41] designed a 19-layer CNN model to classify ODs using fundus images collected based on the International Clinical Diabetic Retinopathy (ICDR) severity scale. According to the ICDR severity scale, these fundus images were classified into five stages of DR. Before training the proposed model, the quality of the fundus images was graded by a senior ophthalmologist. They attained a remarkable specificity of 91.47%.

Farag et al. [42] proposed a DenseNet encoder and a convolutional attention module block-based DL model for DR severity identification. Features were extracted using an encoder from the APTOS 2019 dataset fundus images and then refined using an attention block. Their proposed model showed 82.00% accuracy for the identification of severity DR grading. Vadduri et al. [43] described a method for automatically classifying cases of diabetic eye disease (DED) based on images. Before training the TL models (i.e., Vgg-16, Xception, ResNet-50, CNN), various image enhancement techniques, such as CLAHE and illumination correction, were used. Accuracy levels of 90.00% or higher in the recognition of ODs were achieved by all TL models.

Tan et al. [44] conducted a review to analyze AI methods for the classification of ODs. Their study concluded that the amalgamation of feature extraction methods with DL models was potentially used by researchers for the classification of ODs using retinal imaging. Oliveira et al. [45] proposed a model that uses a visual attention module for the identification of eye-tracking diseases. Their study mainly focused on the diagnosis of autism spectrum disorder (ASD), and they achieved an average precision of 90.00%, a recall of 69.00%, and a specificity of 93.00%. Another study [46] also suggested a model named SGIV for a DCNN for the diagnosis of ASD. Raghavendra et al. [47] designed a novel 18-layer CNN model for the identification of glaucoma using ocular images. For the training of their proposed model, they used a publicly available OD dataset that contains a total of 1426 images, including 589 normal and 837 glaucoma images. They achieved an accuracy of 98.13%.

Ferreira et al. [48] proposed a CNN method for the detection and diagnosis of glaucoma. The proposed CNN model was also used to perform the segmentation of the optic disk. Phylogenetic analysis was utilized to characterize ROIs using texture descriptors. Three databases, including RIM-ONE, DRIONS-DB, and DRISHTI-GS, were used for training and testing the CNN model. Their proposed model outcomes were significant, achieving 100% on all measures (such as recall, specificity, and accuracy) in red channel analysis. Alfifa et al. [49] used a DL architecture with their newly introduced retinal nerve fiber layer (RNFL) to overcome the issue of the shape and size of the optic disk and optic cup. They tested their model on the ORIGA dataset and attained an accuracy of 92.88% with an AUC of 89.34%.

Using a CNN on fundus images, the authors of [50] proposed a two-stage method for OD localization. A semi-automatic ground truth creation strategy was proposed that gives the essential annotations enabling the training of a YOLOv4-based model for autonomous OD localization. They trained their proposed method on the ORIGA dataset for OD localization. They achieved promising results of 87.40%, 89.79%, and 88.70% in accuracy, precision, and recall, respectively. Additionally, one study [51] used OCT images to analyze retinal disorders. Khan et al. [52] designed a CNN model by integrating several optimization methods for the detection of various eye disorders. The performance of the proposed model was tested on several datasets, including ODIR, DR-HAGIS, and IDRiD, and achieved a diagnostic accuracy of 95.5%.

A rule-based NLP model was designed by Wykoff [53] for classifying proliferative diabetic retinopathy (PDR) and non-proliferative diabetic retinopathy (NPDR) severity by using clinical notes. The proposed model achieved notable outcomes such as 98.8% PPV and 90.5% sensitivity. Another study [54] developed a novel model named the novel MTL-based teacher ensemble method based on knowledge distillation for classifying eye diseases. The proposed model was evaluated on a dataset of 7212 labeled and 35,854 unlabeled images across 3502 patients and attained 83% accuracy.

Lu et al. [55] developed a CNN model by combining two deep networks, i.e., ResNet-50 and Vgg-19, for the classification of retinal diseases. The encoder–decoder network model was used to extract the semantic data from OCT images, and the ReLayNet model was used to perform the segmentation of retinal layers from OCT images. Their proposed model obtained the first position in the MICCAI RETOUCH challenge in 2017 on both the segmentation and classification of retinal diseases. For segmenting the retinal layers, the proposed model achieves a dice coefficient of 76.67%, while an AUC of 1.00 was achieved in detecting eye diseases.

Szeskin et al. [56] designed a CNN with a combination of dilated convolution filters to perform the pixelwise classification of OCT scans. The proposed model was tested on 106 clinical OCT scans and yielded an F1-score of 0.78 and an AUC of 0.937 in classifying eye diseases. Another study [57] designed a CNN model and tested it on 1338 retinal glaucoma images. The average scores achieved by the proposed model were 97.04% recall, 98.99% specificity, and 97.20% precision. Devalla et al. [58] designed a novel deep learning network called a dilated residual U-Net (DRU-NET) for segmenting glaucoma. The proposed model achieves a notable outcome of the dice coefficient, which was 0.91 ± 0.05 in the detection of glaucoma disease.

Arslan et al. [59] proposed two CNN models for segmentation by combining SSF-Net and TSF-Net. The performance of the model was evaluated on an open dataset, named Retinal Images for Pigment Signs. The proposed model was executed for up to 4-fold cross-validation. The results reveal that SSF-Net and TSF-Net show significant results for the screening and analysis of retinal diseases. Haider et al. [60] designed two models, SLS-Net and SLSR-Net, for the pixelwise segmentation of an optic cup and optic disk for the identification of glaucoma. They trained and tested their proposed network on four datasets and achieved remarkable outcomes in segmenting the ODs. Furthermore, another study [61] also developed two networks named ESS-Net and FBSS-Net for the segmentation of OD and OC for glaucoma detection and achieved good results.

**Table 1:** Recent studies that used deep learning models for the classification of ODs.

Ref	Year	Method	Dataset Name	No of Diseases	Outcomes
Lenka et al. [62]	2025	GCN	DRISTHI-GS	02	Accuracy = 97.43%
Hu et al. [63]	2025	FundusNet	UKBB and EyePACS	02	AUC = 77.00%
Kansal et al. [64]	2025	TL + LDA + BiLSTM	ODIR	08	Accuracy 98.04%
Butt et al. [65]	2025	CNN	DDR	05	Accuracy = 95.92%
Nguyen et al. [66]	2024	ResNet-152	Eye diseases using UFI	02	Accuracy = 96.47%
Li et al. [67]	2024	CNN	TRIPOD	02	Accuracy = 92.04%
Al-Fahdawi et al. [68]	2024	HRNet	OIA-ODIR	08	Accuracy = 88.56%
Hussain et al. [69]	2024	CNN	OHD	02	Accuracy = 96.15%
Hemelings et al. [70]	2023	CNN	AIROGS	02	Accuracy = 85.84%
Sengar et al. [71]	2023	CNN	RFMiD	02	Accuracy = 90.02%
Thanki [72]	2023	DCNN	DRISTHI-GS	02	Accuracy = 75.30%
Nazir et al. [73]	2021	CNN	EYEPACS datasets	02	Accuracy = 97.13%
Bodapati et al [74]	2021	DCNN	APTOS 2019	01	Accuracy = 84.31%
Khan et al. [75]	2021	VGG-19	APTOS 2019	04	Accuracy = 97.47%
Sarki et al. [76]	2021	CNN	Messidor-2	01	Accuracy = 81.33%
Pahuja et al. [77]	2022	SVM and CNN	APTOS 2019	02	Accuracy = 85.42%
Vidivelli et al. [78]	2025	CNN	ODIR	05	Accuracy = 89.64%
Farag et al. [79]	2022	CBAM	APTOS 2019	02	Accuracy = 93.45%
Vives et al. [80]	2021	CNN	APTOS 2019	02	Accuracy = 94.54%
Zhang et al. [81]	2022	CNN	APTOS 2019	02	Accuracy = 96.15%
Gangwar et al. [82]	2021	ResNet-50	APTOS 2019	02	Accuracy = 92.39%

In previous studies [2,4,9,16,32,33,34,35,36], we observed several limitations, as some studies [62,63,64,65,66,67,68] focused on binary classification, determining whether ODs were present or not. However, a few studies [75,77] addressed multiple ODs but did not achieve satisfactory results. A major reason for this was that the dataset of OD images used by these studies was insufficient and imbalanced. Additionally, large pre-trained models were used by prior studies [72,73,74,77,78,79,80,81,82,83], which led to the issue of gradient vanishing, affecting classification accuracy. To address these challenges, we proposed the ODDM. In this model, we used SM-TOM to balance the imbalanced classes. Additionally, we simplified the ODDM to reduce the number of trainable parameters, which will improve the classification results significantly.

## 3. Materials and Methods

This section presents the experimental methodology for the evaluation of the ODDM with baseline models.

### 3.1. Workflow of ODDM for Classification of ODs

**Figure 2:**
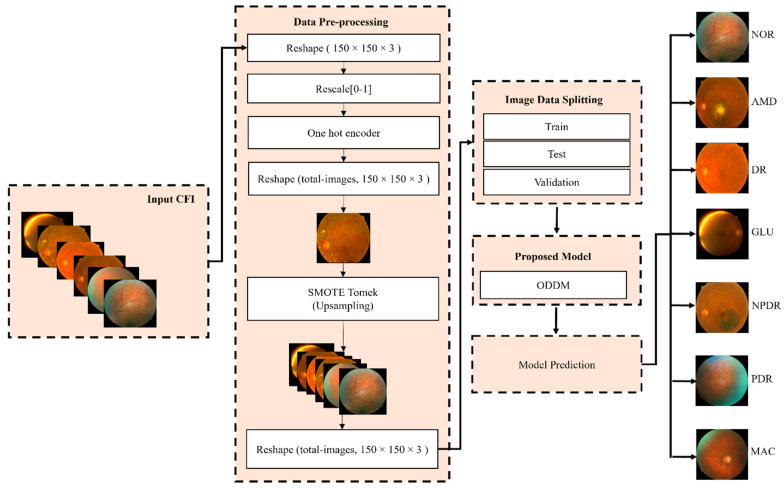
The workflow of the proposed ODDM for the classification of ODs using CFIs.

Diabetes is a major factor in infections of the human eyes. Fundus images are used by researchers to diagnose ODs. The early detection of ODs can protect patients from severe eye complications or blindness. Several studies [81,82,83] used DL algorithms to enhance the accuracy of the detection of ODs. The development of AI and image processing has directly contributed to the substantial transformation that has taken place in the field of medical imaging [84,85,86]. Thus, this study designs an ODDM based on DL for the classification of seven different types of ODs, including NOR, AMD, DR, GL, MP, NPDR, and PDR, using CFIs. We fixed the size of the input CFI to 150 × 150 × 3 to reduce the computational cost. Additionally, data normalization and SM-TOM methods were used to protect the ODDM from overfitting and resolve the issue of an unequal distribution of CFI samples within each class of the dataset [72]. The OD dataset was divided into 4-fold cross-validation sets for training, validation, and testing. The proposed ODDM and seven baseline models were executed up to 30 epochs, and the value of the learning rate is 0.00001. The reason for executing the models up to 30 epochs is that we used early stopping for every 5 epochs and examined the results to ensure that the model remained safe from overfitting. In addition to that, we changed the learning rate whenever the model was overfitted. We trained the model for 50 epochs, but the model’s performance converged, and training more would not significantly improve the results after 30 epochs. The classification performance of the proposed ODDM and other models was compared in terms of many metrics, such as accuracy, loss, precision, recall, area under the curve (AUC), and F1-score. A GRAD-CAM heatmap was produced by using the ODDM to visualize ODs. Figure 2 presents the proposed framework used for the classification of ODs.

### 3.2. Dataset Description

**Figure 3:**
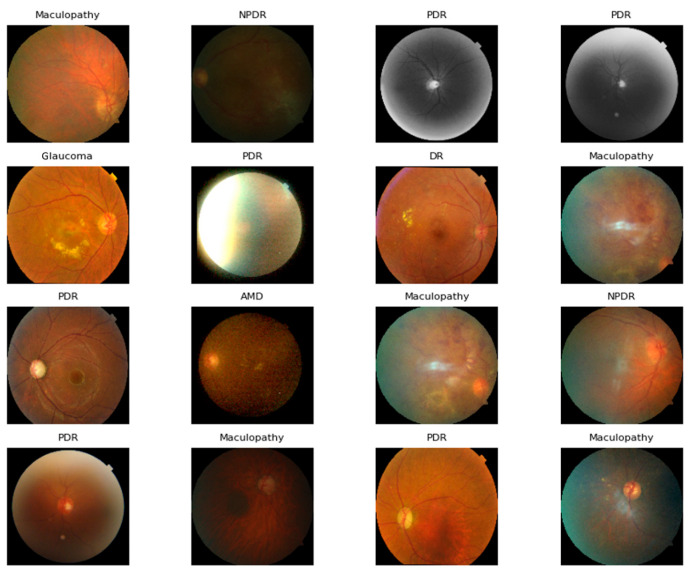
A few original sample images of ocular diseases before using SM-TOM.

In this study, we used the OD dataset created by Cen et al. [25]. This dataset contains CFIs, which correspond to several different ODs from the Joint Shantou International Eye Center (JSIEC), Shantou City, Guangdong Province, China. A total of 2572 images, including 273 CFIs of AMD, 318 CFIs of DR, 270 CFIs of MAC, 368 CFIs of NPDR, 576 CFIs of NOR, 404 CFIs of PDR, and 363 CFIs of GLU, were used to evaluate the proposed ODDM to classify these diseases. Table 2 presents a detailed summary of the dataset. Additionally, Figure 3 depicts sample images of ODs.

### 3.3. Handling Imbalanced Classes of OD Dataset Using SM-TOM

**Figure 4:**
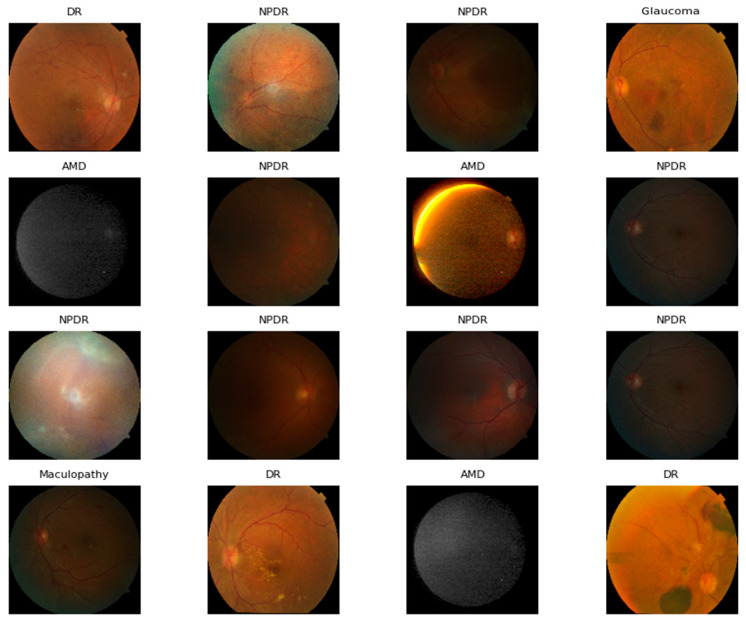
Synthetic CFIs of ODs generated by using SM-TOM.

The dataset used in this study has an imbalanced number of OD images. Several studies [87,88,89,90] conclude that imbalanced datasets can affect the training of the model. Therefore, we applied SM-TOM to balance the OD dataset. The SMOTE oversampling approach was designed by Chawla et al. [89]. The contrasting random sampling method only duplicates the random images from the minority class. SMOTE generates CFIs based on the Euclidean distance of each minority class data point, and Tomek is the modified form of the condensed nearest neighbor, which is also used for increasing the number of image samples of the minority class. We also set the K-nearest neighbors (K = 5) for SM-TOM. This study combines SM-TOM to generate different synthetic images from the original image data, as depicted in Figure 4. Additionally, the pseudocode of the proposed SM-TOM is outlined in Algorithm 1. A detailed summary of the OD dataset after applying SM-TOM is presented in Table 3.
**Algorithm 1:** SMOTE Tomek algorithm for increasing the number of CFI of the minority class.Input: S→ Set for training, M→ instances of minority set, U→ No of nearest neighbors,C→ Quantity of synthetic CFI images to compensate the original CFI in the minority classes of ODs.Output: A group of synthetic samples from the minority: Oˈ1: ST= ɸ    // ST **is a collection of samples that are generated using Smote Tomek.**$2:\;\mathrm{For}\;\mathrm{all}\;O_{i}$ in O  do: $\;\;\;\;\;\;\;\;\;\;\;\;\;\;\;\;\;\;\;\;\;\;\;N_{oi}$ ← k$\;\mathrm{nearest}\;\mathrm{neighbors}\;\mathrm{of}\;O_{i}$ in S                        n$\;\leftarrow \;\mathrm{the}\;\mathrm{number}\;\mathrm{of}\;\mathrm{samples}\;\mathrm{in}\;N_{oi}$  and not in O                        if k/2 ≤ n <k$\;\mathrm{then}\;\;\;\;\;\;\;\;\;\;\;\;\;\;\;\;\;\;\;\;\;\;\;\;\;\;\;//o_{i}$  **is a borderline sample.** $\;\;\;\;\;\;\;\;\;\;\;\;\;\;\;\;\;\;\;\;\;\;\;\mathrm{add}\;o_{i}\;to\;ST$
                    **End** if

**End**
3: Oˈ= ɸ                       //Oˈ  **is a set containing synthetic samples.****4: For all**$\;\;{stʹ}_{i}$  **in** ST  **do:**
             **For** i=1 to C  do:                o ← choose a random sample from $N_{sti}$ ${\;\;\;\;\;\;\;\;\;\;\;\;\;\;\;stʹ}_{i}$ ← ${stʹ}_{i}$  + j * (${stʹ}_{i}-o)$ is a random number in (0, 1), ${stʹ}_{i}$ is a synthetic CFI.
                      **add**
${stʹ}_{i}$  to Oˈ
                      **End** For **End** For**5:**Oˈ = O U Oˈ6:      returnOˈ

### 3.4. K-Fold Cross-Validation

For this study, we applied K-fold Validation (CV) where K = 4. In 4-fold CV, the ODDM was trained on three folds and tested on one independent fold. Additionally, the ODs used in the testing fold were not utilized in the testing phase. A detailed summary of the dataset after applying SM-TOM with 4-fold CV is presented in Table 4.

### 3.5. Proposed ODDM

**Figure 5:**
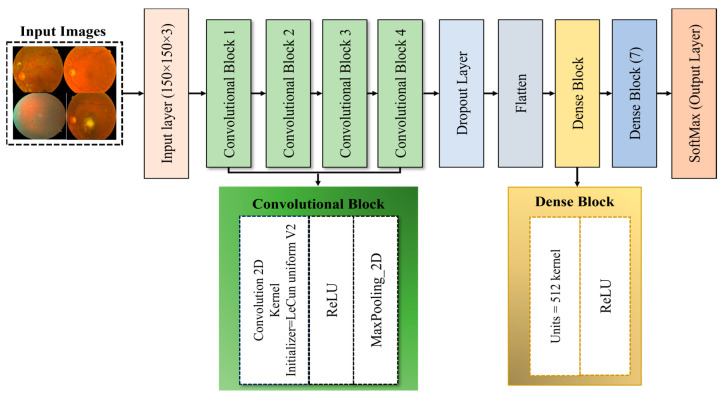
The structure of the proposed ODDM used for the classification of seven different ODs using CFIs.

DL methods have widely been used by recent studies [88,89,90] for the diagnosis of several diseases, such as skin cancer [91], COVID-19 [92], breast cancer [93,94,95], etc. This study designs a custom model named the OD detection model (ODDM), which is based on a CNN. The purpose of the ODDM is to classify seven ODs, including NOR, AMD, DR, GLU, MAC, NPDR, and PDR, using CFIs. The proposed ODDM consists of five convolutional blocks (ConvL_Bs), non-linear activation functions (N_LAFs), fully connected layers (FCLs), a dropout layer (D_PL), and dense layer blocks (D_LB). Each ConvL_B takes three steps (i.e., convolution2D (Conv_2D), ReLU, and max pooling (M_PL)) to complete the process. Additionally, D_LB is based on units of 512 kernels and the ReLU function. The proposed ODDM is illustrated in Figure 5. Table 5 presents a detailed summary of the proposed ODDM and its training parameters.

#### 3.5.1. ConvL_Bs of ODDM

The primary element of the proposed ODDM consists of three steps: (a) kernel-based convolution, (b) stacking, and (c) the use of N_LAFs to complete the process. Therefore, the LecunUniformV2 initializer was used to assign the kernel. For this study, we fixed the input image to 150 × 150 × 3 and set the kernel size to 5 × 5.

Consider the input matrix $I_{M}$, kernels $K_{u}$, ∀l ∈[1,…,L], and an output O, (here output O means the output of the entire three-step process). For an individual kernel $K_{u}$, the convolution output is calculated by using Equation (1).

(1) $$\mathrm{Step}\;1:\;f\left(l\right)=I_{M}\;⊗\;K_{u},\;\forall l\;\in [1,\ldots ,L]$$

where ⊗ represents a convolution operation. Then, all $f\left(l\right)$ matrixes are stacked into a three-dimensional matrix M, as discussed in Equation (2).

(2) $$\mathrm{Step}\;2:\;M=[f\left(1\right),\ldots ,f(L)]$$

Finally, the matrix M is passed into the N_LAFs and outputs the final matrix.

(3) Step 3: O=N_LAF(M)

This study computes the sizes A of three main components (input, kernel, and output), as described in Equation (4).

(4) $$A\left(y\right)=\left\{\begin{array}{cc} \;\;\;\;\;W_{I\;\;}\times H_{I}\times C_{I}\; & \;\;\;\;\;\;\;\;\;\;\;\;\;\;\;\;\;\;\;\;\;\;\;\;\;\;\;\;\;\;\;\;\;\;\;\;\;\;\;\;y=I_{M}\;\\ \;\;\;\;\;\;W_{K}\times H_{K}\times C_{K} & \;\;\;\;\;\;y=K_{u},\;\forall l\;\in [1,\ldots ,L]\;\;\;\;\;\;\;\\ \;\;\;\;\;\;W_{j}\times H_{j}\times C_{j}\; & \;\;\;\;\;\;\;\;\;\;\;\;\;\;\;\;\;\;\;\;\;\;\;\;\;\;\;\;\;\;\;\;\;\;\;\;\;\;\;\;\;\;y=J \end{array}\;\right.$$

The above-mentioned triple elements $\left(W_{I},\;H_{I},\;C_{I}\right)$ represent the size of the width, height, and channels of the matrix, respectively [30]. The subscripts I, K, and J represent input, kernel, and output, respectively. L denotes the total number of filters. Note that $C_{I}=C_{K}$, which means that the channel of input $C_{I}$ should equal the channel of the kernel $C_{K}$. Supposing that these filters move with the padding of $n_{p}$ and stride of $n_{s}$, we can obtain the sizes $\left(W_{j}\times H_{j}\;\times C_{j}\right)$ of output matrix J, as presented in Equation (5).

(5) $$\left\{\begin{array}{c} W_{J}=1+\frac{\left(2\times n_{p}+W_{I}-W_{K}\right)}{n_{s}}\\ H_{J}=1+\frac{\left(2\times n_{p}+H_{I}-H_{K}\right)}{n_{s}}\\ C_{J}=L \end{array}\right.$$

Here the channel of output $C_{J}$ should equal the number of filters L.

#### 3.5.2. Flatten Layer

For this study, we place the flattened layer (F_LT) between ConvL_B and D_LB. The purpose of the F_LT layer is to convert 2D data into a 1D array, and then this data is provided to the FCL of the proposed ODDM. The purpose of using the F_LT layer in the ODDM is to flatten the spatial dimension data of the CFI. Equation (6) is used to perform the F_LT process.

(6) $$F_{F\_LT}=Flatten\;(A_{y})$$

#### 3.5.3. D_LB of Proposed ODDM

The N_LAFs are used in D_LB, and details about them are presented below. For this study, we used ReLU as N_LAF, which is represented by P. Supposing that $R_{ST}$ is the entry of the matrix M, we used Equations (7) and (8) to perform ReLU operations.

(7) $$P_{ReLU}\left(R_{ST}\right)=ReLU(R_{ST})$$

(8) $$ReLU(R_{ST})=max\;(0,R_{ST})$$

The dense layer takes in a single matrix and produces results based on that matrix’s attributes. Using a dense layer, the output of the proposed ODDM involves classifying the data into their respective classes. Therefore, the SoftMax activation method is used in the dense layer for this classification. SoftMax is a probability-based activation function where the total number of classes is equal to the number of neurons. For this study, the number of classes and neurons is 7.

Equation (9) is used to perform the SoftMax operation.

(9) $$S_{i}=\frac{e^{R_{{ST}_{i}}}}{\sum_{j}^{n}e^{R_{{ST}_{j}}}}$$

Additionally, the proposed ODDM contains a total of 1,091,495 parameters, of which 1,091,495 are trainable parameters, and none of the parameters are non-trainable.

### 3.6. Performance Evaluation

The performance of the proposed ODDM was measured by using a confusion matrix. For this study, 4-fold cross-validation was used for the training and validation of the proposed ODDM and baseline models. The proposed ODDM and baseline models were evaluated in terms of several metrics, as discussed in Equations (10)–(14).

(10) $$Accuracy=\frac{TP+TN}{TP+FN+FP+TN}$$

(11) $$Precision=\frac{TP}{TP+FP}$$

(12) $$Recall=\frac{TP}{TP+FN}$$

(13) $$F1-Score=2\times \frac{Precision\times Recall}{Precision+Recall}$$

(14) $$MCC=\frac{\left(TP\times TN-FP\times FN\right)}{\sqrt{\left(TP+FP)(TP+FN)(TN+FP)(TN+FN\right)}}$$

### 3.7. ANOVA and Tukey’s HSD Post Hoc Test

For this study, an ANOVA (analysis of variance) [95] test is applied to determine the statistically significant difference between the proposed ODDM and seven models, including the R_1_, R_2_, R_3_, R_4_, R_5_, R_6_, and R_7_ models. However, the ANOVA test only presents the difference that exists in the models, not where the difference lies. Therefore, Tukey’s HSD post hoc [96] test is also used to compare the models pairwise. This study considers two hypotheses: (1) the Null Hypothesis (H_0_) and (2) Alternative Hypothesis (H_1_). H_0_ indicates that there is no significant difference in the accuracy obtained by the proposed ODDM and other baseline models. Additionally, H_1_ suggests that at least one model has a significant difference compared to other models in terms of accuracy.

### 3.8. Proposed Algorithm

In this study, the pseudocode of the proposed ODDM is presented in Algorithm 2. The structure of Algorithm 2 is divided into 05 sections, namely $\left[Z_{1},Z_{2},Z_{3},Z_{4},Z_{5}\right]$. The pre-processing of the CFIs of ODs is discussed in $Z_{1}$. The process of balancing the size of the CFIs by using the SM-TOM method is presented in $Z_{2}$. The architecture of the proposed ODDM is provided in $Z_{3}$. After enhancing the size of the CFI dataset, the training and validation process of the proposed ODDM is discussed in $Z_{4}$. Finally, the performance of the proposed ODDM is computed in $Z_{5}$.
**Algorithm 2**:Classification of ocular diseases using CFI.**Input**:$A_{1}$= CFI**Output**:Ocular Diseases Classification**PRE-PROCESSING: Z_1_****1**$Z_{1}:A_{1}\to A_{3}$**2**$Rescale\;Image:\;A_{1}\to A_{2}$**3**$Normalization:\;A_{2}\to A_{3}$**SYNTHETIC IMAGES USING SM-TOM: Z_2_****4**$A_{3}\to$ See **Algorithm (1)****PROPOSED ODDM MODEL: Z_3_****5**$Z_{3}\to O\left(1\right):$         **For** *i* in $O\left(1\right):$
                       **Add** Conv_2D in $O\left(1\right)$ See **Equation** (5)                        **Add** ReLU in $O\left(1\right)$ See **Equations** (7) and (8)                        **Add** M_PL in $O\left(1\right)$ See **Equations** (1)–(4)         **End**
**Add** F_LT in $Z_{3}$ See **Equation** (6) **Add** D_BL in $Z_{3}$         **For**
*j* in D_BL:                       **Add** ReLU in D_BL See **Equations** (7) and (8)                       **Add** SoftMax in D_BL See **Equation** (9)         **End** **End****TRAINING & VALIDATION SPLIT FOR ODDM MODEL: Z_4_****6****Training set**: $A^{Train\_OD\_CFI}\left(i\right)$, Validation set: $A^{Val\_OD\_CFI}\left(i\right)$**7****For** f = 1 : |$A^{Train\_OD\_CFI}\left(i\right)$ on A_3_**8**               **Training Image**: $A^{Train\_OD\_CFI}\left(i,r\right)$**9**${\;\;\;\;\;\;\;\;\;\;\;\;\;\;\;A}^{Train\_OD\_CFI}\left(r\right)$: training CFI image in epoch runs *(r)***10**${\;\;\;\;\;\;\;\;\;\;\;\;\;\;\;A}^{Train\_OD\_CFI}\left(r\right)\;\to \;A^{Train\_OD\_CFI}\left(i,r\right)$**11****End****12**$Pred\;\left(t,\;u\right)=predict\;[H\left(t,u\right),\;{\;A}^{Train\_OD\_CFI}\left(i\right)\;]$**PERFORMANCE EVALUATION PARAMETERS: Z_5_****13****For** Z = 1:5% Z represents the no. of performance evaluators.                **Parameters**: $P^{m}\left(t,u\right)$ See **Equations** (10)–(14) **End****14****Select** Best Model ${B(P}^{m})$ in terms of Z**15****End**

## 4. Results and Discussions

This section presents the comprehensive outcomes obtained by using the proposed ODDM with and without SM-TOM and other baseline models such as R_1_, R_2_, R_3_, R_4_, R_5_, R_6_, and R_7_.

### 4.1. Experimental Setups and Hyperparameters of Proposed ODDM and Baseline Models

In this study, TensorFlow (TF) v2.16.1 was used for the implementation of the proposed ODDM and baseline models. Additionally, the Keras library was also used with TF to perform the backend process and execution of the proposed ODDM. The imbalanced-learn library v0.12.3 was used for the implementation of the SM-TOM method. For this work, the operations that are not associated with neural networks were programmed using the Python language version 3.13.0. The entire experiment was executed on a workstation equipped with a Windows 10 operating system, which has specifications including a Core i8 processor of the 11th generation, 32 GB of RAM, and an 11 GB NVIDIA GPU. Table 6 presents the hyperparameters that are used to fine-tune the proposed ODDM.

### 4.2. Results of Proposed ODDM and Baseline Models

The performance of the proposed ODDM with and without using SM-TOM is compared with baseline models in classifying ODs using CFIs. Additionally, the comprehensive results obtained by using the proposed ODDM are shown in Table 7.

#### 4.2.1. Results of Proposed ODDM in Terms of Accuracy

**Figure 6:**
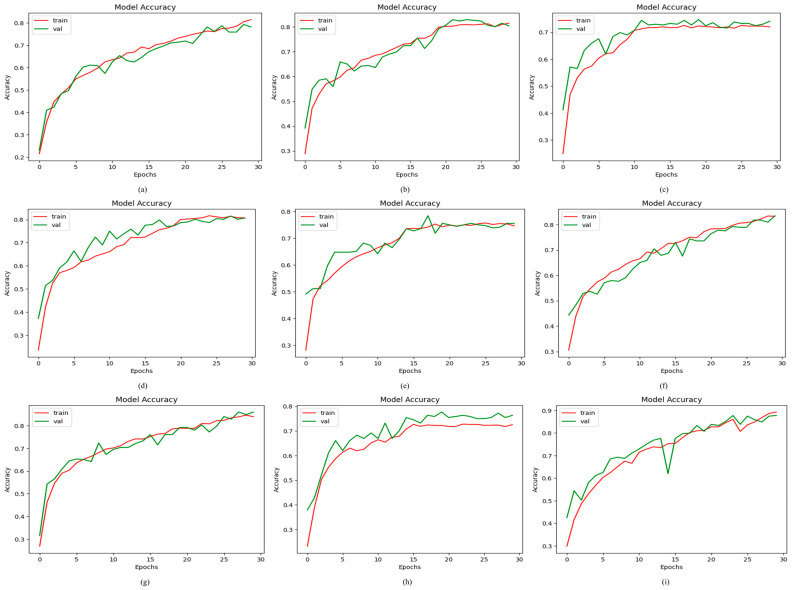
Results achieved by proposed ODDM and baseline models in terms of accuracy: (**a**) R_1_, (**b**) R_2_, (**c**) R_3_, (**d**) R_4_, (**e**) R_5_, (**f**) R_6_, (**g**) R_7_, (**h**) proposed ODDM without SM-TOM, and (**i**) proposed ODDM with SM-TOM.

From Table 7, it is observed that the proposed ODDM with SM-TOM achieves the highest classification accuracy of 97.19% (95% CI (Confidence Interval): 95.50–98.80%), precision of 95.23%, recall of 88.74%, F1-score of 88.31%, and AUC of 98.94%. The models R_5_ and R_6_ achieve a classification accuracy of 73.33% (95% CI: 68.91–77.81%) and 85.14% (95% CI: 81.60–88.70%), respectively. The lowest result obtained by R_3_ in terms of accuracy is 73.13% (95% CI: 68.70–77.60%). Additionally, R_2_ and R_7_ achieve a classification accuracy of 80.80% (95% CI: 76.80–84.40%) and 83.15% (95% CI: 79.40–87.00%), respectively. Furthermore, the proposed model without SM-TOM attains a classification accuracy of 77.15% and an F1-score of 75.12%. Figure 6 presents the graphical representation attained by using the proposed ODDM and other models in terms of accuracy.

#### 4.2.2. Results of Proposed ODDM in Terms of AUC

**Figure 7:**
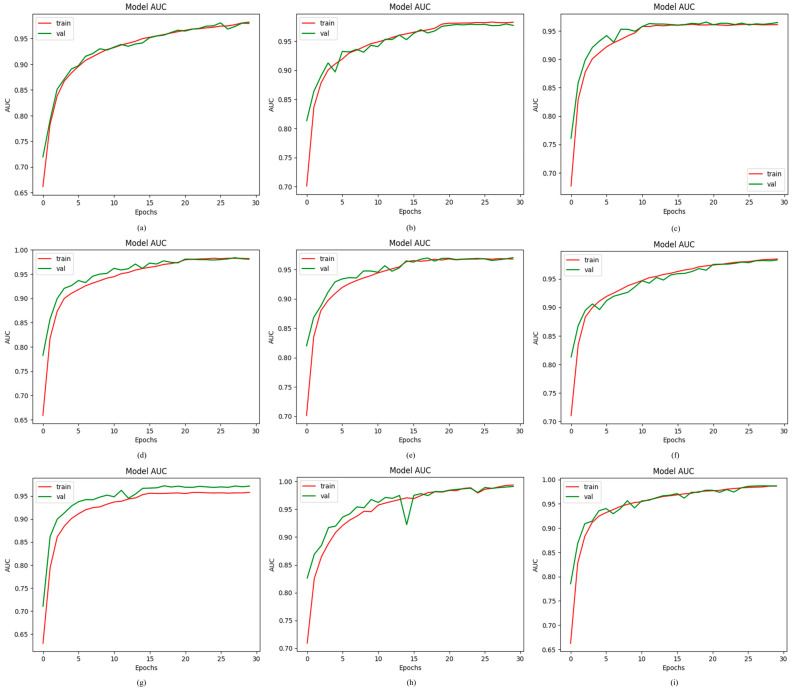
Results achieved by proposed ODDM and baseline models in terms of AUC: (**a**) R_1_, (**b**) R_2_, (**c**) R_3_, (**d**) R_4_, (**e**) R_5_, (**f**) R_6_, (**g**) R_7_, (**h**) proposed ODDM without SM-TOM, and (**i**) proposed ODDM with SM-TOM.

This study uses the AUC to calculate the efficacy of the proposed ODDM in distinguishing OD classes. The value of the AUC ranges between 0 and 1. A higher value shows that the model performs significantly well in classifying the seven ODs, including NOR, AMD, DR, GLU, MAC, NPDR, and PDR. Therefore, to observe their efficiency, a comprehensive comparison was made between the proposed ODDM and the baseline models. The proposed ODDM with SM-TOM attains the highest AUC of 98.94%. R_1_, R_6_, R_2_, R_5_, R_4_, R_7_, and R_3_ attain an AUC of 98.46%, 98.57%, 97.85%, 96.66%, 98.17%, 98.12%, and 96.04%, respectively. Additionally, the AUC of 96.31% is achieved by the proposed ODDM without SM-TOM. The results show that the performance of the proposed ODDM with SM-TOM is high compared to baseline models in terms of the AUC. The outcomes of these models are illustrated in Figure 7.

#### 4.2.3. Results of Proposed ODDM in Terms of Precision

**Figure 8:**
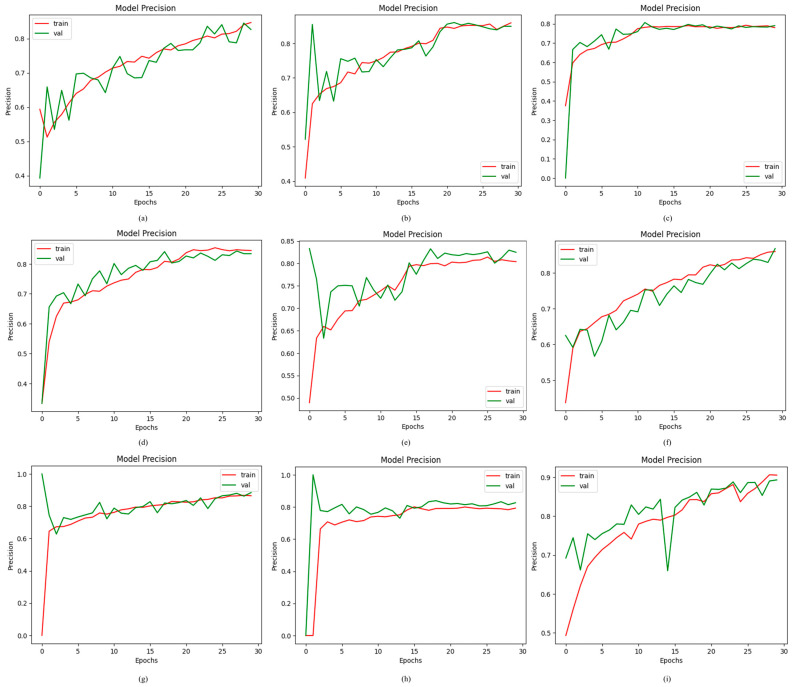
Results achieved by proposed ODDM and baseline models in terms of precision: (**a**) R_1_, (**b**) R_2_, (**c**) R_3_, (**d**) R_4_, (**e**) R_5_, (**f**) R_6_, (**g**) R_7_, (**h**) proposed ODDM without SM-TOM, and (**i**) proposed ODDM with SM-TOM.

The precision metric is used to measure the TP prediction obtained by the model in classifying ODs using CFIs. A greater value of precision means that the models used in this study predict a lower value of FP. A graphical representation of the results obtained by the proposed ODDM and baseline models is depicted in Figure 8. The results reveal that the proposed ODDM with SM-TOM achieves the highest precision of 95.23% as compared to other models. Moreover, the proposed ODDM without SM-TOM achieved a precision of 83.73%. R_1_ attains a precision of 86.58%. The other models R_6_, R_4_, R_2_, R_7_, and R_3_ attain a precision of 88.66%, 82.74%, 83.77%, 84.63%, and 80.39%, respectively. The lowest precision value is attained by R_5_, which is 79.23%.

#### 4.2.4. Results of Proposed ODDM in Terms of Recall

**Figure 9:**
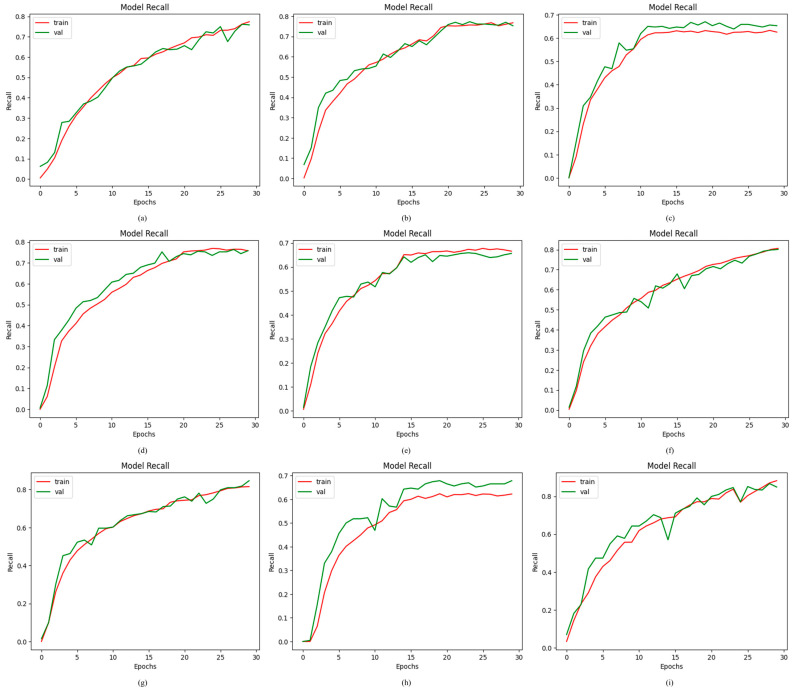
Results achieved by proposed ODDM and baseline models in terms of recall: (**a**) R_1_, (**b**) R_2_, (**c**) R_3_, (**d**) R_4_, (**e**) R_5_, (**f**) R_6_, (**g**) R_7_, (**h**) proposed ODDM without SM-TOM, and (**i**) proposed ODDM with SM-TOM.

The purpose of recall is to accurately identify TPs from the actual positive CFIs of the OD dataset used in this work. Figure 9 illustrates the recall curve that is generated by evaluating the proposed ODDM in comparison to R_1_, R_5_, R_6_, R_3_, R_2_, and R_4_. The highest recall value of 88.74% is achieved by the proposed ODDM with SM-TOM. The lowest recall value of 66.13% is attained by R_3_. The models R_7_, R_6_, R_1_, R_4_, R_2_, and R_5_ achieved a recall of 81.01%, 83.02%, 79.41%, 75.46%, 75.73%, and 66.13, respectively.

#### 4.2.5. Results of Proposed ODDM in Terms of F1-Score

**Figure 10:**
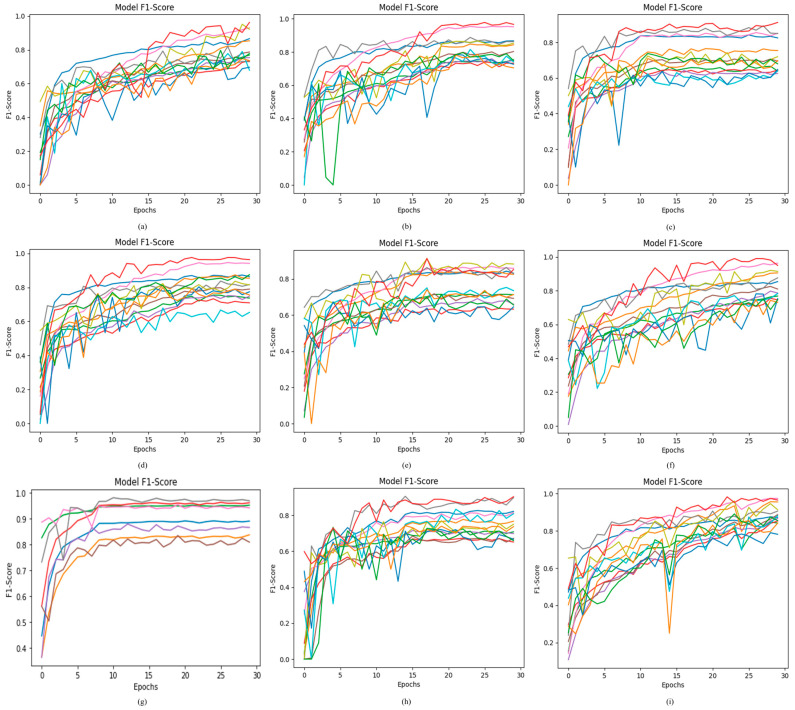
Results achieved by proposed ODDM and baseline models in terms of F1-score: (**a**) R_1_, (**b**) R_2_, (**c**) R_3_, (**d**) R_4_, (**e**) R_5_, (**f**) R_6_, (**g**) R_7_, (**h**) proposed ODDM without SM-TOM, and (**i**) proposed ODDM with SM-TOM.

The F1-score is used to measure the harmonic mean of precision and recall. A greater value of the F1-score means that the model performs appropriately in classifying the seven classes of ODs. The proposed ODDM with SM-TOM attains the highest F1-score of 88.31% as compared to the other models used in this study. R_5_ and R_6_ achieve an F1-score of 74.19% and 84.94%, respectively. The R_1_ model achieves an F1-score of 83.18%. The F1-score for R_4_ and R_2_ is 78.91% and 81.04%, respectively. R_3_ attained the lowest F1-score at 73.05%. The detailed results obtained by using the proposed model and baseline models are presented in Figure 10.

#### 4.2.6. Results of Proposed ODDM in Terms of Loss

**Figure 11:**
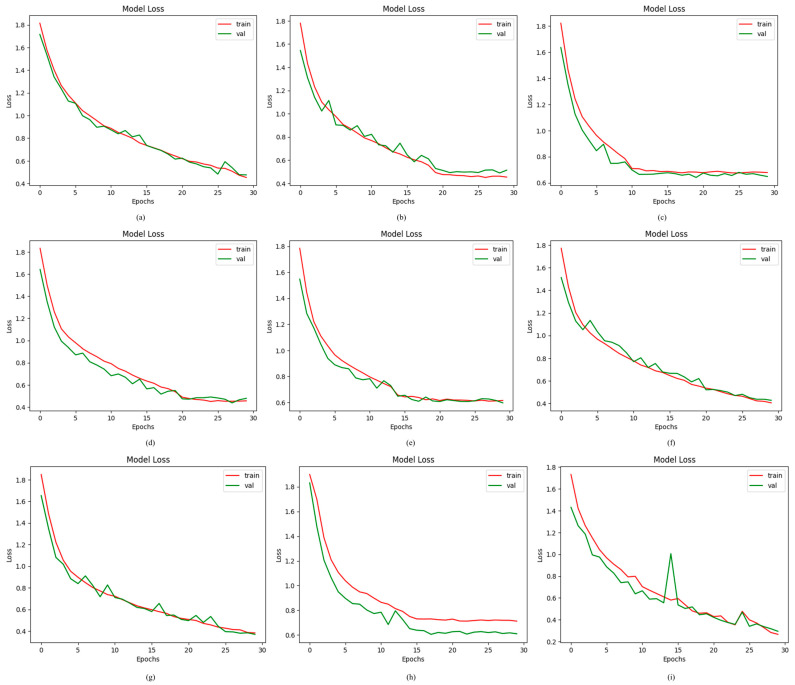
Results achieved by proposed ODDM and baseline models in terms of loss: (**a**) R_1_, (**b**) R_2_, (**c**) R_3_, (**d**) R_4_, (**e**) R_5_, (**f**) R_6_, (**g**) R_7_, (**h**) proposed ODDM without SM-TOM, and (**i**) proposed ODDM with SM-TOM.

Loss functions represent the numerical difference between the predicted and actual values. In this study, the loss of the proposed ODDM and baseline models is determined by using a categorical cross-entropy function. When the proposed ODDM was trained using SM-TOM, the results were remarkable. R_1_ had a loss value of 0.4227%. R_2_ and R_6_ produced loss values of 0.4894% and 0.4005%, respectively. Additionally, R_7_, R_4_, R_5_, and R_3_ achieved loss values of 0.4403, 0.4603%, 0.6267%, and 0.6824%, respectively. The proposed ODDM with SM-TOM produced a loss of 0.3873%. However, the proposed ODDM without SM-TOM generated a loss of 0.6883%. The loss values produced by the proposed ODDM and other models used in this work are presented in Figure 11.

#### 4.2.7. Results of Proposed ODDM in Terms of ROC

**Figure 12:**
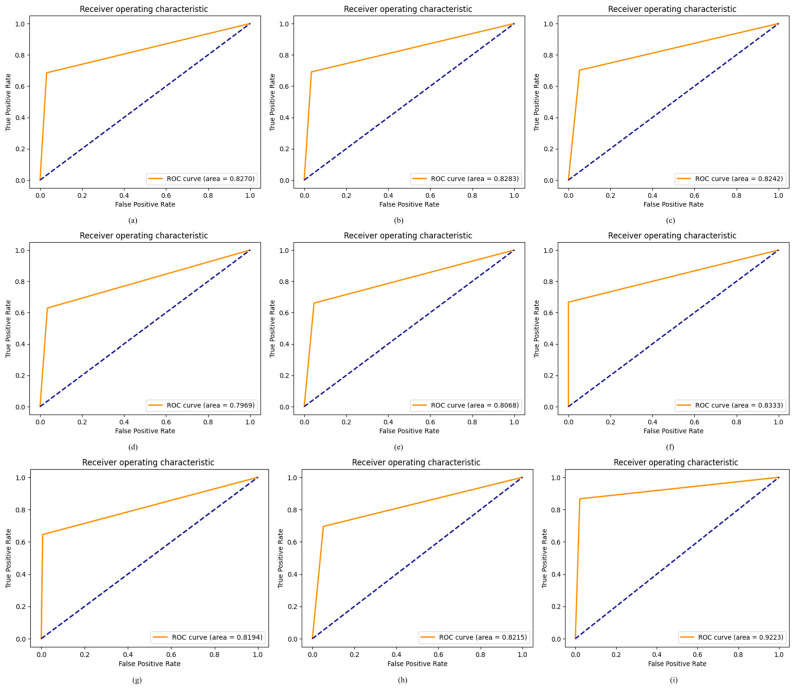
Results achieved by proposed ODDM and baseline models in terms of ROC: (**a**) R_1_, (**b**) R_2_, (**c**) R_3_, (**d**) R_4_, (**e**) R_5_, (**f**) R_6_, (**g**) R_7_, (**h**) proposed ODDM without SM-TOM, and (**i**) proposed ODDM with SM-TOM.

Model efficacy is assessed using an ROC curve, where a larger ROC signifies a more successful model in classifying ODs. The ROC curve was used to compare the proposed ODDM with and without SM-TOM with baseline models after enhancing the OD datasets. The proposed ODDM with and without SM-TOM attained ROC values of 0.9923 and 0.8215, respectively. The baseline models R_1_, R_2_, R_3_, R_4_, R_5_, R_6_, and R_7_ attained ROC values of 0.8290, 0.8283, 0.8242, 0.7969, 0.8068, 0.8333, and 0.8194, respectively, as shown in Figure 12. In the ROC curve, a significant enhancement is observed in the performance of the proposed ODDM, with SM-TOM, which is depicted in Figure 12.

#### 4.2.8. Results of Proposed ODDM in Terms of AU ROC

**Figure 13:**
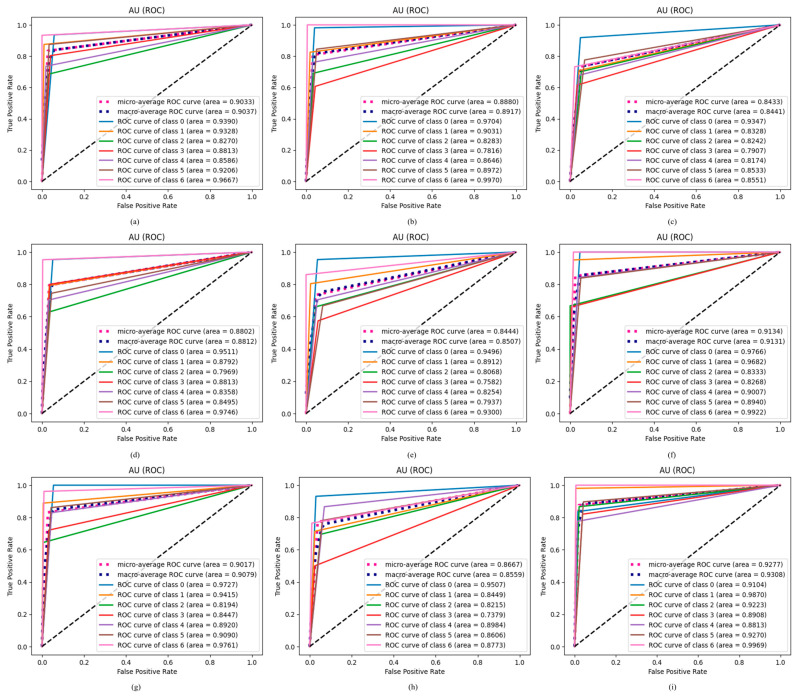
Results achieved by proposed ODDM and baseline models in terms of AU ROC: (**a**) R_1_, (**b**) R_2_, (**c**) R_3_, (**d**) R_4_, (**e**) R_5_, (**f**) R_6_, (**g**) R_7_, (**h**) proposed ODDM without SM-TOM, and (**i**) proposed ODDM with SM-TOM.

In this study, we used AU ROC to perform a classwise evaluation of the proposed ODDM and baseline models. Figure 13 represents the AU ROC of the proposed ODDM and baseline models. In AU ROC, class 0 denotes AMD, class 1 denotes DR, class 2 represents GLU, class 3 represents MAC, class 4 represents NOR, class 5 represents NPDR, and class 6 denotes PDR. The proposed ODDM with SM-TOM achieves the highest micro-average ROC curve of 0.9277 and macro-average ROC curve of 0.9308 as compared to other baseline models. A detailed classwise representation of the proposed ODDM and baseline models is depicted in Figure 13.

#### 4.2.9. Confusion Matrix of Proposed ODDM

**Figure 14:**
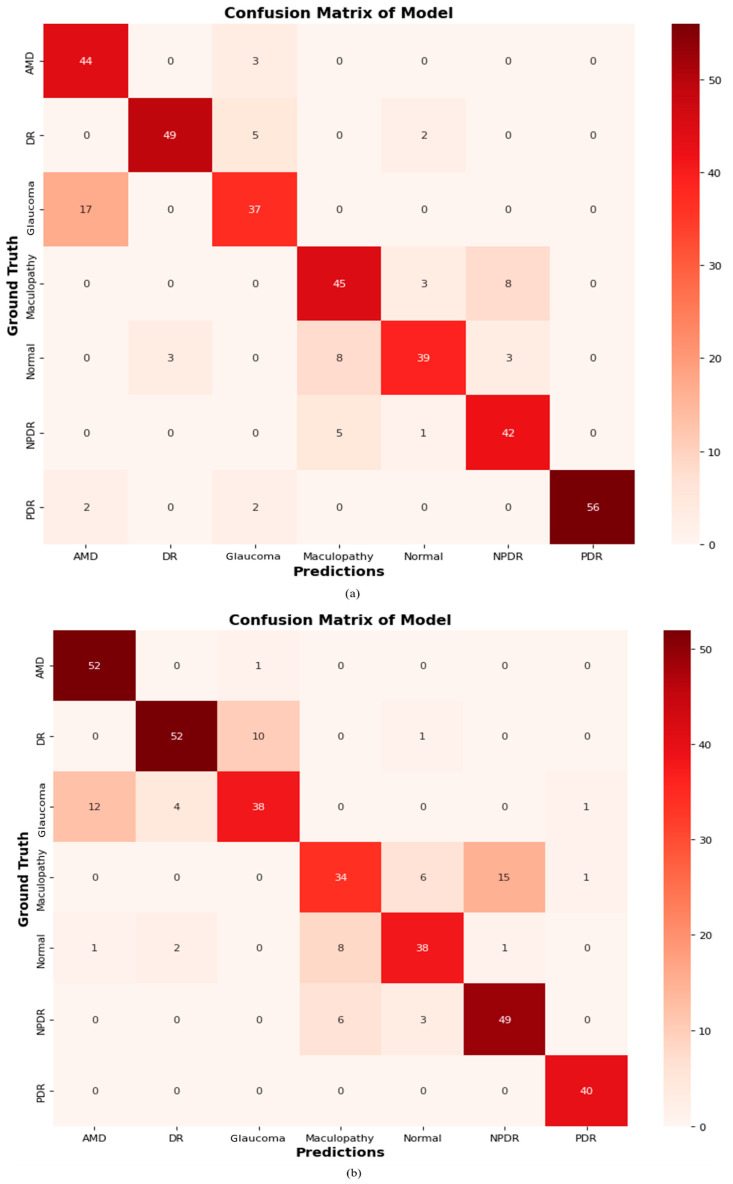

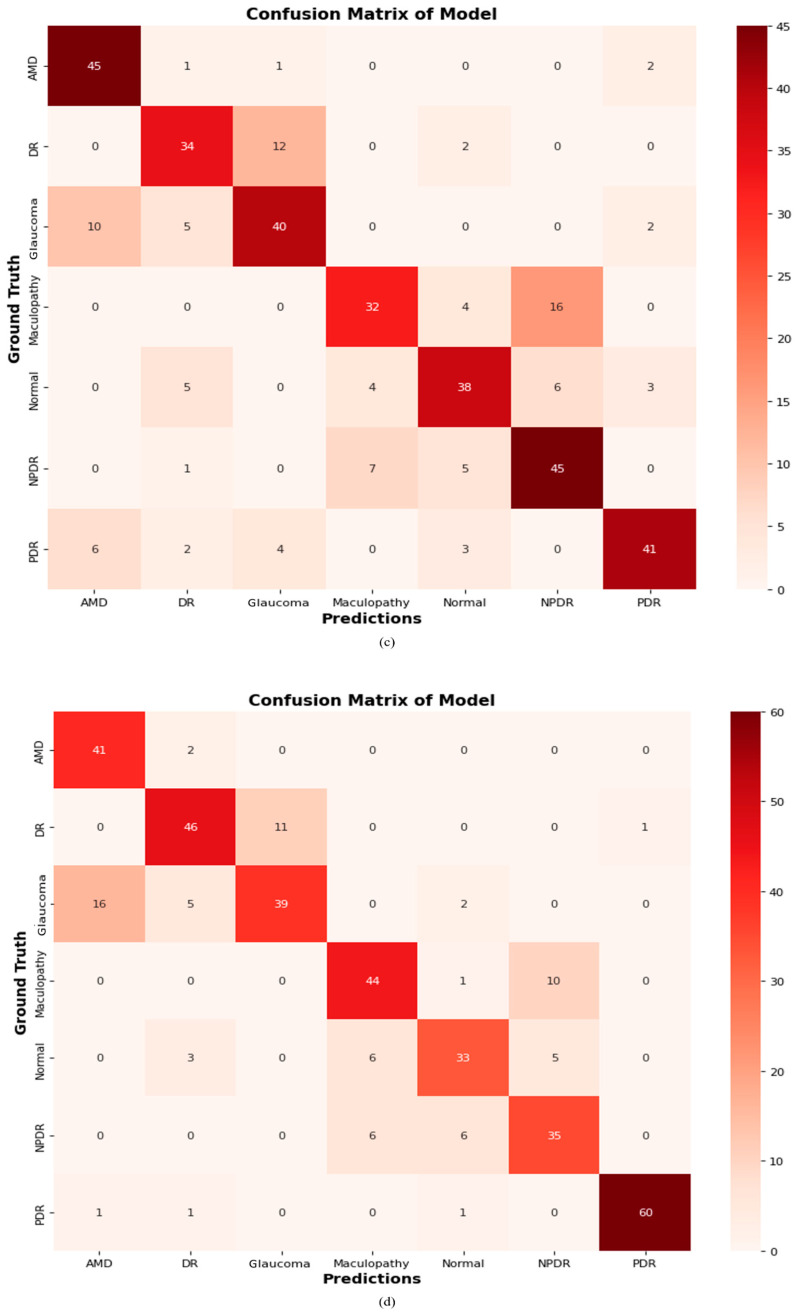

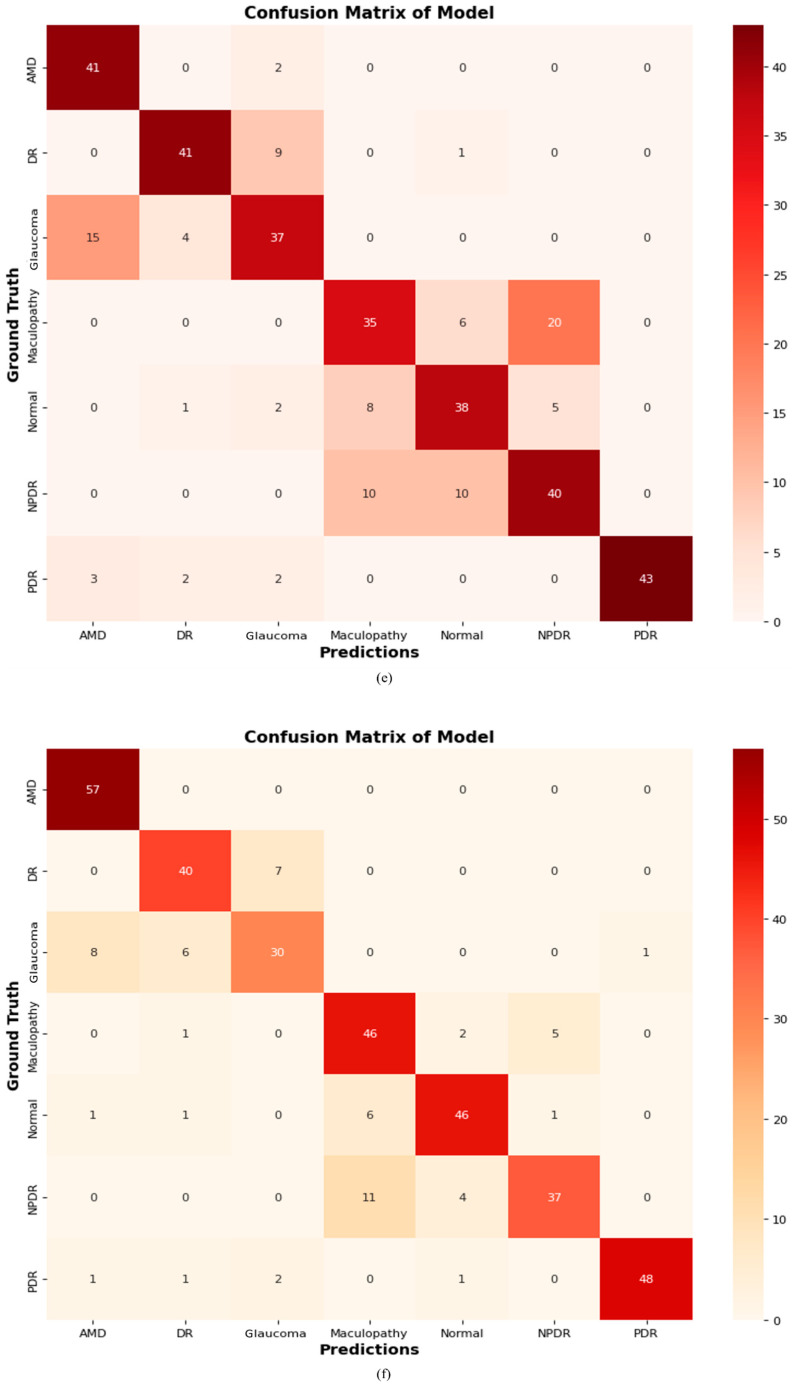

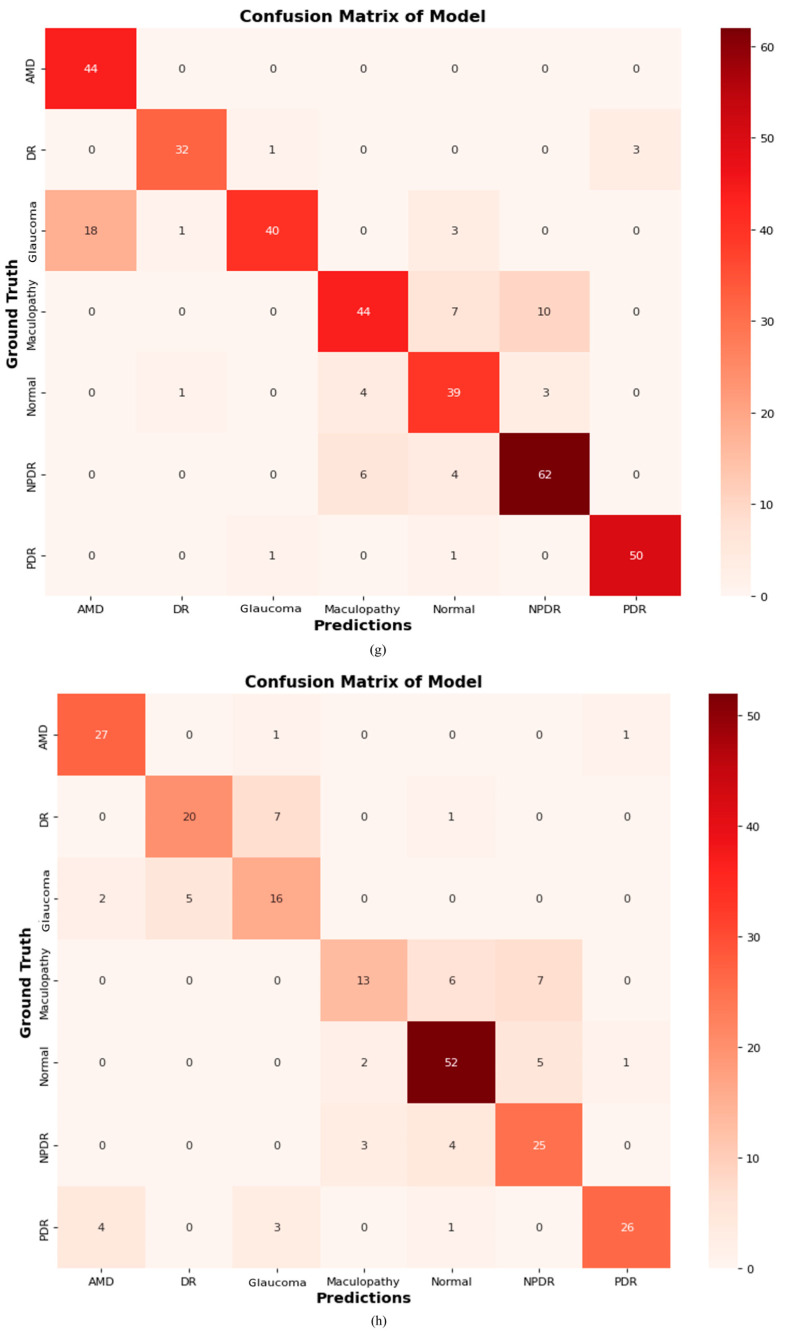

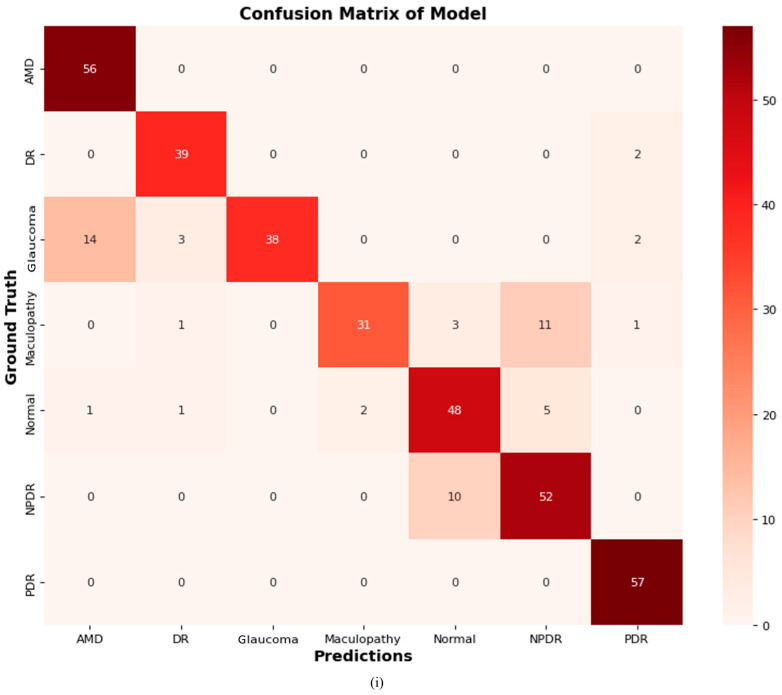
Confusion matrix: (**a**) R_1_, (**b**) R_2_, (**c**) R_3_, (**d**) R_4_, (**e**) R_5_, (**f**) R_6_, (**g**) R_7_, (**h**) proposed ODDM without SM-TOM, and (**i**) proposed ODDM with SM-TOM.

A confusion matrix is generated to evaluate the performance of the proposed ODDM and baseline models, as depicted in Figure 14. The proposed ODDM correctly classifies 56 cases as AMD, 39 cases as DR, 38 cases as GLU, 31 cases as MAC, 48 cases as NOR, 52 cases as NPDR, and 57 cases as PDR. Additionally, the proposed ODDM misclassifies 02 cases of DR as PDR and 03 cases of MAC as NOR. The R_1_ model accurately classifies 44, 49, 37, 45, 39, 42, and 56 cases as AMD, DR, GLU, MAC, NOR, NPDR, and PDR, respectively. Furthermore, R_5_ correctly classifies 41 cases as AMD, 41 cases as DR, 37 cases as GLU, 35 cases as MAC, 38 cases as NOR, 40 cases as NPDR, and 43 cases as PDR. The detailed results of the confusion matrix are presented in Figure 14.

#### 4.2.10. GRAD-CAM Visualization of Proposed ODDM

**Figure 15:**
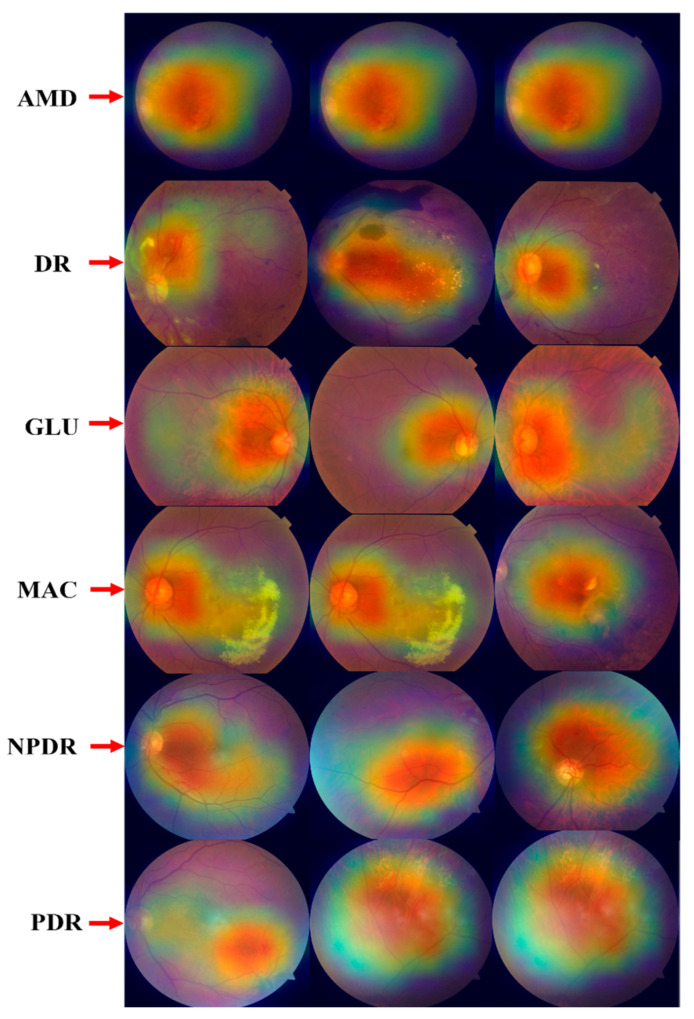
GRAD-CAM visualization of proposed ODDM to highlight infected region.

This section presents the GRAD-CAM approach to visually represent the output of the ODDM. The purpose of the heatmap is to depict the relevant area of the ODs that the proposed ODDM focuses on. Figure 15 illustrates the heatmap of the proposed ODDM.

### 4.3. Ablation Experiments

This work integrates SM-TOM with the proposed ODDM for the classification of ODs using CFIs. To assess whether the proposed ODDM was effective in ODs using CFIs, the outcomes of the ablation experiment were statistically determined using the control variable approach. Table 8 shows the effectiveness of the proposed ODDM with and without SM-TOM in classifying the ODs by using CFIs. In ablation, two experiments were performed. Experiment 1 was used to apply the proposed ODDM for the classification of ODs. Experiment 2 used the proposed ODDM with SM-TOM to classify the ODs.

When comparing the results of Experiments 1 and 2, it is revealed that combining SM-TOM with the ODDM increases the average classification accuracy by 20.04%. Experiment 2 shows noteworthy outcomes that can be attributed to two factors: first, Tomek link [97] undersampling removes samples from majority class boundaries; second, SMOTE [91,92,93] creates synthetic CFIs of the minority class to balance the OD dataset. The proposed ODDM with SM-TOM effectively generalizes across both groups using a balanced OD dataset, leading to improved performance and reduced bias towards the majority class.

### 4.4. Results of ANOVA and Tukey’s HSD Post Hoc Test

This study employs an ANOVA and Tukey’s HSD post hoc test to evaluate the statistical significance of the models. The results obtained by using an ANOVA are presented in Table 9.

From Table 9, it was observed that the *p*-value < 0.05, which rejects H_0_. Thus, this means that there is a statistically significant difference in the accuracy of at least one model compared to other models. We then applied Tukey’s HSD post hoc test to compare the models pairwise. The reason for using Tukey’s HSD post hoc test is to determine which models differ significantly from each other. All detailed results are presented in Table 10.

From Table 10, it was observed that the proposed ODDM (with SM-TOM) statistically outperforms R_1_ with a *p*-value of 0.005. This shows that the proposed ODDM (with SM-TOM) is statistically superior to the R_1_ model. The proposed ODDM is also compared with all other models, including R_2_, R_3_, R_4_, R_5_, R_6_, and R_7_, and the results reveal that the proposed ODDM is statistically significant in all of them.

### 4.5. Comparison of Proposed ODDM with SOTA

This section presents a comparison of the proposed ODDM with recent SOTA methods. Table 11 shows an extensive comparison of the proposed ODDM with SOTA in terms of accuracy.

### 4.6. Discussion

ODs are generally classified by using CFIs [9]. Several studies [11,12,13,14,15,16,17,21,23] used CFIs for the classification of several types of ODs such as DR, AMD, MAC, etc. ODs are clusters of multiple eye diseases, and it is difficult for eye specialists or ophthalmologists to identify the eye disease accurately [26,27,28]. However, the condition of eye diseases varies from patient to patient [30]. Several studies [32,35,36,37,38,39,44,46] conclude that a patient with diabetic history is at risk of diabetic retinopathy. If ODs are not diagnosed in their initial stage, patients can face severe eye complications and blindness. Thus, the objective of this study was to develop a custom model named the ODDM for the classification of seven types of ODs. A publicly available benchmark OD dataset [25] was used. The dataset contains an imbalanced number of images in each class of ODs, as discussed in Table 2. To handle this issue, the SM-TOM method was used to balance the distribution of the CFI in each class of the OD dataset. After applying SM-TOM [46], a detailed enhanced dataset was created and is presented in Table 3. Additionally, Algorithm 1 presents the pseudocode of SM-TOM. Several pre-processing methods were also used. The workflow of the proposed framework is presented in Figure 2. The proposed ODDM was trained and tested on the enhanced dataset. Additionally, the operation of the proposed ODDM is presented in Algorithm 2.

The proposed ODDM with and without employing SM-TOM was compared with seven CNN-based baseline models, including R_1_, R_2_, R_3_, R_4_, R_5_, R_6_, and R_7_. The highest classification accuracy of 97.19% was achieved by the proposed ODDM with SM-TOM. The other seven baseline models R_7_, R_5_, R_6_, R_1_, R_4_, R_2_, and R_3_ produced an accuracy of 83.15%, 73.33%, 85.14%, 83.42%, 79.46%, 80.80%, and 73.13%. Additionally, 77.15% accuracy was achieved by the proposed ODDM without using SM-TOM for the classification of ODs. Table 7 presents the comprehensive results obtained by using the proposed ODDM and other baseline models. Ablation experiments were also performed to observe the effectiveness of the proposed ODDM with SM-TOM, as discussed in Table 8. Additionally, ANOVA and Tukey HSD post hoc tests were also performed to represent the statistical significance of the proposed model and other models used in this work for the classification of ODs. The results in Table 10 demonstrate that the proposed ODDM with SM-TOM is statistically superior compared to the R_1_, R_2_, R_3_, R_4_, R_5_, R_6_, and R_7_ models.

With a significant classification accuracy of 97.19%, the outcomes shown in Table 7 demonstrate the effectiveness of the proposed ODDM with SM-TOM in classifying ODs and extracting prominent discriminative patterns from CFIs. The baseline models used in this study attained less classification accuracy due to their deep network [52], and their last convolutional layer limited their classification performance [93,94,95]. The filter size of these baseline models was not appropriate [55], and their neurons connected to the input layer are so large that they may neglect the dominant features of the CFI. Furthermore, a vanishing gradient [58,59,60,61,62,63,64,65,66,67,68] problem occurred throughout the training process of these baseline models. The integration of SM-TOM and the proposed ODDM resolves all of these issues. In the proposed ODDM, a streamlined CNN layer structure is included to keep the gradient from disappearing. Furthermore, the proposed ODDM generates fewer training parameters, which further lowers the network’s complexity, as discussed in Table 5.

As discussed in Table 11, Al-Fahdawi et al. [68] proposed a CNN-based deepNet model for the classification of OD. They achieved a classification accuracy of 74.62%. Vidivelli et al. [37] proposed a model named CataractNetDetect for the classification of cataracts from pairs of CFIs. The CataractNetDetect model achieves a 97.90% AUC. Another study [97] proposed a CNN model for the diagnosis of ODs. This proposed model produced an accuracy of 72.70%. Rubina et al. [39] used a Vgg-16 model for the classification of DR. Vgg-16 achieved a classification accuracy of 85.95% on mild multi-class DED. Farag et al. [42] proposed a CBAM model for the identification of the severity level of diabetic retinopathy and achieved an accuracy of 95.00%. Pawar et al. [41] designed a 19-layer CNN model to classify ODs using the CFIs obtained from ICDR. The proposed 19-layer CNN model attained a remarkable specificity of 91.47%. Bhati et al. [93] used a CNN model for ophthalmic disease detection on imbalanced fundus images. They achieved a classification accuracy of 93.14%. Vadduri et al. [43] applied various TL methods for automatically classifying DED based on images. Accuracy levels of 90.00% or higher in classifying DED were achieved by all TL models. This study proposed an ODDM with SM-TOM for the classification of ODs by CFIs on an imbalanced dataset. The proposed ODDM achieved a classification accuracy of 97.17%, which is superior to that of SOTA classifiers.

## 5. Conclusions

ODs represent a group of diseases that affect the functioning of the human eye. CFIs are used to diagnose ODs. Therefore, this study designed the proposed ODDM with SM-TOM for the classification of ODs using CFIs. The proposed ODDM contains convolutional blocks with further layers, such as ConvL, max pooling, and ReLU, to classify ODs. The ocular dataset contains an imbalanced distribution of images in each class of ODs, so SM-TOM was used to handle this problem. Additionally, Grad-CAM shows a heatmap of class activation to demonstrate the process of the proposed ODDM. The proposed ODDM with SM-TOM obtained an accuracy of 97.19%, a recall of 88.74%, a precision of 95.23%, a 98.94% AUC, and an F1-score of 88.31%, which were superior to those of baseline models as well as modern SOTA. Ablation experiments also demonstrated the effectiveness of the proposed ODDM with SM-TOM. Thus, this study concluded that the proposed ODDM with SM-TOM can be a great help to ophthalmologists in classifying ODs such as AMD, DR, MAC, NPDR, PDR, GLU, and NOR. One limitation of this study is that the proposed ODDM is not suitable for the OCT images used for the identification of ODs. In the future, we will integrate the federated learning model with the proposed ODDM for patient data privacy.

## Figures and Tables

**Table 2:** A summary of the ocular disease dataset before applying SM-TOM.

No. of Ocular Classes	Ocular Diseases	No. of CFIs
0	AMD	273
1	DR	318
2	MAC	270
3	NPDR	368
4	NOR	576
5	PDR	404
6	GLU	363
Total		2572

**Table 3:** A summary of the ocular disease dataset after applying SM-TOM.

No. of Ocular Classes	Ocular Diseases	No. of CFI
0	AMD	576
1	DR	576
2	MAC	576
3	NPDR	576
4	NOR	576
5	PDR	576
6	GLU	576
Total		4032

**Table 4:** A summary of the ocular disease dataset after applying SM-TOM and 4-fold CV.

Folds	AMD	DR	MAC	NPDR	NOR	PDR	GLU	Total
1	144	144	144	144	144	144	144	1008
2	144	144	144	144	144	144	144	1008
3	144	144	144	144	144	144	144	1008
4	144	144	144	144	144	144	144	1008
Total	576	576	576	576	576	576	576	4032

**Table 5:** A comprehensive summary of the proposed ODDM.

Layer Type	Output Shape	Parameters
Con_conv2d_(Conv2D)	(None, 146, 146, 16)	1216
MPL_average_pooling2d_(AveragePooling2D)	(None, 73, 73, 16)	0
Con_conv2d_1_(Conv2D)	(None, 69, 69, 32)	12,832
MPL_average_pooling2d_1_(AveragePooling2D)	(None, 34, 34, 32)	0
Con_conv2d_2_(Conv2D)	(None, 30, 30, 64)	51,264
MPL_average_pooling2d_2_(AveragePooling2D)	(None, 15, 15, 64)	0
Con_conv2d_3_(Conv2D)	(None, 11, 11, 128)	204,928
MPL_average_pooling2d_3_(AveragePooling2D)	(None, 5, 5, 128)	0
DO_dropout_(Dropout)	(None, 5, 5, 128)	0
FLT_flatten_(Flatten)	(None, 3200)	0
D_Dense_(Dense)	(None, 512)	819,456
DO_dropout_1_(Dropout)	(None, 512)	0
D_dense_1_(Dense)	(None, 7)	1799
Total params:		1,091,495
Trainable params:		1,091,495
Non-trainable params:		0

**Table 6:** The hyperparameters of the proposed ODDM.

Hyperparameters	Value
Learning rate	0.00001
Batch size	32
Momentum	0.9
No. of iterations	30 epochs
Activation function	ReLU, SoftMax
Optimizer	RMSprop

**Table 7:** Evaluation of proposed ODDM and baseline models in terms of many performance metrics.

Models	Accuracy (%)	Precision (%)	Recall (%)	F1-Score (%)	AUC (%)
R_5_	73.33	79.23	66.13	74.19	96.66
R_6_	85.14	88.66	83.02	84.94	98.57
R_1_	83.42	86.58	79.41	83.18	98.46
R_4_	79.46	82.74	75.46	78.91	98.17
R_2_	80.80	83.77	75.73	81.04	97.85
R_3_	73.13	80.39	64.36	73.05	96.04
R_7_	83.15	84.63	81.01	82.32	98.12
Proposed ODDM (Without SM-TOM)	77.15	83.73	66.81	75.12	96.31
Proposed ODDM (With SM-TOM)	97.19	95.23	88.74	88.31	98.94

**Table 8:** Outcomes produced by proposed ODDM with and without SM-TOM for classification of ODs using CFIs.

Experiments	Proposed Model	SM-TOM	Image Size	Accuracy
1	ODDM	×	150 × 150 × 3	77.15%
2	ODDM	✓	150 × 150 × 3	97.19%

**Table 9:** Results obtained by using ANOVA test.

Test	F-Statistics	*p*-Value
ANOVA	17.31	0.0021

**Table 10:** Pairwise comparison of proposed model with baseline models for measuring statistical significance in terms of accuracy.

Comparison	Mean Difference	*p*-Value	Statistically Significant?
Proposed ODDM (With SM-TOM) vs. R_1_	6.96	0.005	Yes
Proposed ODDM (With SM-TOM) vs. R_2_	8.18	0.004	Yes
Proposed ODDM (With SM-TOM) vs. R_3_	12.02	0.003	Yes
Proposed ODDM (With SM-TOM) vs. R_4_	8.85	0.042	Yes
Proposed ODDM (With SM-TOM) vs. R_5_	11.92	0.003	Yes
Proposed ODDM (With SM-TOM) vs. R_6_	6.01	0.001	Yes
Proposed ODDM (With SM-TOM) vs. R_7_	7.02	0.002	Yes

**Table 11:** Comparison of proposed ODDM with SOTA.

Ref	Year	Models	No. of ODs	Ocular Diseases	Accuracy
[78]	2025	CNN		GLU, DR, Hypertension, Myopia, and Cataract	89.64%
[94]	2025	Attention Module	3	Myopia, Normal, and Other Ocular Diseases	90.40%
[95]	2025	Attention Module	2	Multiple Ophthalmology	95.30%
[96]	2024	Attention with Inception-V3	4	NOR, DME, CNV, and Drusen	96.00%
[68]	2024	Deep-Net	5	DR, AMD, Hypertension, GLU, and Cataract	74.62%
[97]	2024	CNN	2	GLU and Normal	72.70%
[42]	2022	CBAM	2	DR and Normal	95.00%
[81]	2022	TL	2	DR and Normal	91.20%
[82]	2021	CNN	2	DR and Normal	82.18%
[70]	2023	CNN	2	GLU and Normal	92.50%
[93]	2023	CNN	3	DR, Cataract, and GLU	93.14%
[73]	2023	CNN	2	GLU and Normal	83.00%
Proposed ODDM with SM-TOM			7	DR, AMD, MAC, GLU, NPDR, PDR, and NOR	97.19%

## Data Availability

The original contributions presented in this study are included in the article. Further inquiries can be directed to the corresponding authors.

## Abbreviations

The following abbreviations are used in this manuscript:

ODs	Ocular Diseases
CFIs	Color Fundus Images
DL	Deep Learning
CNN	Convolutional Neural Network
NOR	Normal
AMD	Age-Related Macular Degeneration
DR	Diabetic Retinopathy
GLU	Glaucoma
MAC	Maculopathy
NPDR	Non-Proliferative Diabetic Retinopathy
PDR	Proliferative Diabetic Retinopathy
